# Associations between adverse childhood experiences and trust in health and other information from public services, professionals and wider sources: national cross sectional survey

**DOI:** 10.1136/bmjph-2023-000868

**Published:** 2024-05-27

**Authors:** Mark A Bellis, Karen Hughes, Kat Ford, Catherine Sharp, Rebecca Hill

**Affiliations:** 1 Liverpool John Moores University, Liverpool, UK; 2 Policy and International Health, WHO Collaborating Centre on Investment for Health and Well-being, Public Health Wales, Wrexham, UK; 3 College of Medicine and Health, Bangor University, Wrexham, UK

**Keywords:** Community Health, Public Health, Cross-Sectional Studies, Preventive Medicine

## Abstract

**ABSTRACT:**

**Introduction:**

Trust in health and other systems can affect uptake of public health advice and engagement with health services. Individuals who had adverse childhood experiences (ACEs) are more likely to experience ill health at earlier ages. Ensuring their engagement with health and other services is important in improving their life course prospects, but little is known about how ACEs affect trust in such services and the information they provide.

**Methods:**

Data were collected via a national household survey of residents in Wales (aged ≥18 years, n=1880, November 2022–March 2023). Questions measured ACE exposure and trust in health, social, police, charities and government, and health and general information provided by a variety of professionals and sources.

**Results:**

Individuals with ACEs were more likely to report low trust in health advice from hospital doctors, general practitioners (GPs), nurses, pharmacists, and NHS 111, an online and telephone urgent care service (eg, adjusted low trust prevalence: GPs, 0 ACEs 5.3%, ≥4 ACEs 10.4%; NHS 111, 0 ACEs 11.9%, ≥4 ACEs 24.1%). Low trust in services also increased with ACEs, with low trust in police being 3.8 times more likely with ≥4 ACEs (vs 0 ACEs). The highest adjusted prevalence of low trust in a service was for government, rising from 48.4% (0 ACEs) to 73.7% (≥4 ACEs). Low trust in general advice and information from TV/radio programmes rose from 17.6% (0 ACEs) to 30.1% (≥4 ACEs); low trust in social media was higher with an equivalent rise from 61.6% to 75.6%.

**Conclusion:**

Breaking intergenerational cycles of ill health and inequity requires engaging and influencing those with ACEs. However, a history of ACEs was associated with lower trust in supporting institutions, systems and professionals. Empirical data on which resources are most trusted by those with ACEs should be used to facilitate better communications with this vulnerable group.

**WHAT IS ALREADY KNOWN ON THIS TOPIC**

- A history of adverse childhood experiences (ACEs) is a common feature in many national populations, with greater exposure to ACEs increasing risks of poor health outcomes across the life course.
- Although individuals with ACEs could benefit substantively from health and social advice and support, little research has examined which systems, services and professionals those with ACEs distrust and if trust preferences differ from those with no ACEs.

**WHAT THIS STUDY ADDS**

- Results provide empirical information that ACEs are associated with lower levels of trust in health, social, criminal justice, government and other systems and professionals, as well as online sources of health and other information.

**HOW THIS STUDY MIGHT AFFECT RESEARCH, PRACTICE OR POLICY**

- A better understanding of which sources of advice and support are distrusted and trusted by those with a history of ACEs should inform messaging, outreach and other forms of service engagement with this vulnerable group.

## Introduction

 Adverse childhood experiences (ACEs) include child physical, verbal and sexual maltreatment as well as exposure to other potential sources of trauma, such as growing up in domiciles with domestic violence, mental ill health and substance misuse.^1^ Exposure to ACEs is consistently associated with low educational attendance and attainment, school suspension and expulsion, and reduced employment prospects.^2^,^5^ ACEs are also linked to the adoption of health harming behaviours, such as smoking, alcohol misuse, illicit drug use and sexual risk taking^6^; involvement in antisocial behaviours, crime and violence^7 8^; and poorer diets and obesity.^9 10^ Across the life course, higher levels of ACE exposure are predictive of poorer mental health^6 11^ and associated with earlier development of non-communicable disease, including cancer, cardiovascular disease, and subsequent premature mortality.^6 12 13^ Increasingly biological and epigenetic markers of ill health and premature ageing are being disproportionately identified in those exposed to ACEs, indicative of causal relationships.^14^,^16^ ACEs are a common feature of populations in high, middle and low income countries, and predict poorer life course outcomes across each economic level.^17^,^19^ A meta-analysis of data from 206 ACE studies in 22 countries found that 60% of adults had been exposed to at least one ACE while growing up, with 16% exposed to four or more ACEs.^17^ Consequently, preventing ACEs and mitigating their potential impacts are major public health considerations as well as factors of substantial economic importance, with the health burden attributable to ACEs having been estimated to cost over US$1 trillion per annum across North America and Europe alone.^20^

Individuals who have suffered ACEs may benefit from health promotion information that, for instance, advises reductions in substance use or adherence to health guidelines and encourages healthier diets and exercise. Equally, health protection advice on safer sex, routine infection, biometric screening and vaccine uptake is likely to be especially pertinent to those with ACEs, and therefore at higher risks of infection or ill health.^6 15 21^ Early engagement with health services when ill health develops is also a predictor of better outcomes^22 23^ and likely to be especially important to those with ACEs who may develop chronic disease earlier in life. However, studies suggest that exposure to ACEs can influence individuals’ likelihood of: adopting health advice on behaviour change;^24^ vaccination uptake;^25^ engaging in screening programmes which might lead to earlier health and social interventions;^26 27^ and complying with medication and other clinical treatment regimens.^28^ Recent pandemic conditions highlighted poorer uptake of both health protection advice and COVID-19 vaccination in those with ACEs,^29^ increasing risks of infection and transmission.

A major factor associated with adoption of public sector advice and successful engagement with health, social and other support services is levels of trust in sources and providers.^30^,^32^ Moreover, with commercial websites, charities, TV, radio, peers and other sources often providing information independent of the public sector sources, relative trust in public sector versus other commercial, charity and social media outlets is an important consideration, especially when trying to reach and support vulnerable groups.^33^ However, relatively little is known about how ACEs may impact levels of trust in health advice, public and other systems, or general sources of health and other information.

Here, we used data from a national survey to examine levels of trust in different public, private, social and charitable agencies and professionals. We tested the hypothesis that levels of low trust will vary according to the number of ACEs experienced. Based on the findings, we considered which individuals, agencies and communication platforms are best placed to engage and influence those with a history of ACEs.

## Materials and methods

A national household survey of residents in Wales (aged ≥18 years) was conducted between November 2022 and March 2023. A methodology using face-to-face surveys at participants’ places of residence was chosen as this has provided higher levels of compliance in previous ACE studies than, for instance, telephone based surveys.^34^ Moreover, data on location of domicile (used in residence based surveying) provides an ecological measure of deprivation that can be used in stratified sampling. Such data may not be available when sampling through telephone number lists. Sampling and data collection were undertaken by a professional market research company (MRC). To ensure adequate capture of individuals with exposure to multiple ACEs (≥4, n~200), a target sample of 2000 participants was set based on ACE prevalence rates in previous national surveys.^35^ A stratified quota sampling methodology was used to obtain a sample representative of national sociodemographics. The sampling unit was lower super output areas (LSOAs; geographical units with a population of approximately 1500 people), with sampling stratified by Welsh Health Board and deprivation quintile (using Welsh index of multiple deprivation (WIMD) 2019 scores).^36^ WIMD is a combination of eight separate domains of deprivation (income, employment, health, education, access to services, community safety, physical environment and housing), with each domain compiled from a range of different indicators.

A proportionate sample of 200 LSOAs was randomly selected from each deprivation quintile in each health board. A target of 10 interviews was set for each selected LSOA, with quota samples by age and sex. Residential addresses within selected LSOAs were identified using the postcode address file. Only one individual per household was eligible to participate, with study inclusion criteria being age ≥18 years, resident in a selected LSOA and cognitively able to participate.

Interviewers were a mix of men and women, all employed by the MRC. Each was trained to ensure they understood the purpose and content of the survey, with training including information on ACEs and the need for sensitivity and objectivity when undertaking interviews. Households were visited by interviewers and, on contact, potential participants were provided with a participant information sheet and a letter of authority from Public Health Wales. Interviewers detailed: the purpose of the survey and how findings would be used; survey content; and the voluntary, confidential and anonymous nature of the survey. All study materials were provided in Welsh and English language, and participants were able to complete the survey in the language of their choice. All participants provided informed consent, recorded electronically within the survey. Face-to-face interviews were conducted at the door via computer assisted personal interviewing with computer assisted self-interviewing for more sensitive questions (eg, ACE questions). Participation took an average of 22 min, following which all participants were provided with a thank you leaflet containing information for appropriate national support services. Just under half (49%) of all individuals contacted agreed to participate in the survey (n=2007).

### Study questionnaire

The study questionnaire included questions on trust in health advice and general information from various sources, and overall trust in health services, social services, police, government and charities; exposure to ACEs before the age of 18 years; and a range of demographics. Online supplemental table A1 provides the questions used to measure ACEs and outcome measures included in this study.

Guidelines for measuring trust recommend a scale of 0 to 10.^37^ Consequently, trust in health advice for each item was measured by the question “On a scale of 0 to 10 where 0 is not at all and 10 is completely, how much would you trust health advice given to you by: GPs (general practitioners); hospital doctors; nurses; pharmacists/chemists (hereafter termed pharmacists); health professionals available through NHS 111 (hereafter termed NHS 111); health professionals accessed via other means (eg, an app; hereafter termed virtual health professionals); and friends, family or colleagues”. NHS 111 is an online and telephone urgent care health service which provides advice and signposting to appropriate information and services. Trust in service/systems was measured by the question “On a scale of 0 to 10 where 0 is not at all and 10 is completely, how much do you trust: health services; social services; police; charities/voluntary organisations (hereafter termed charities); and government”. Trust in general advice/information was measured by the question “On a scale of 0 to 10 where 0 is not at all and 10 is completely, how much do you trust general advice and information from: NHS websites; health apps for smartphones or tablets (hereafter termed health apps); other internet sites/internet searches (eg, Google, YouTube, Wikipedia; hereafter termed general internet sites); social media (such as Twitter and Facebook); and TV/radio programmes”. Guidelines for measuring trust also include the use of thresholds for categorising trust levels.^37^ However, with no specific threshold for low trust stipulated, for all outcomes we adopted ratings below the midpoint of the scale, <5, as having low trust. This consistent approach across outcomes also assisted with comparisons of low trust percentages between different sources of advice and information. For all trust measurements, those who were unable to provide a rating were recorded as ‘don’t know/not applicable’.

An adapted version of the Centers for Disease Control and Prevention short ACE tool^38^ was used to measure exposure to nine ACE types: verbal, physical and sexual abuse; parental separation/divorce; witnessing domestic violence; and living within a household where mental illness, alcohol or drug misuse was present, or where a household member was incarcerated. Out of the nine types of ACEs, the number individuals reported was summed to generate an ACE count (0 ACEs, 1 ACE, 2–3 ACEs, ≥4 ACEs).

Participants’ sex, age and ethnicity (UK census categories) were collected. Age was re-categorised into four age groups (18–29, 30–49, 50–69 and ≥70 years). For the purpose of analysis and due to low levels in other than white ethnicities, ethnicity was categorised into white and other than white. Residential deprivation was measured using the WIMD quintile (1=most deprived, 5=least deprived) for LSOA of residence.

### Statistical analysis

For the purpose of analyses, the sample was restricted to participants who had complete demographic and ACE count data, for a final sample of 1880. Statistical analyses were conducted in SPSS V.24. χ^2^ tests were used to measure bivariate relationships for all outcome measures and ACE counts and participant demographics. Binary logistic regression (enter method) was used to measure independent associations between ACEs and all outcomes, controlling for participant age, sex, ethnicity and residential deprivation quintile. Generalised linear modelling was used to generate estimated marginal means (adjusted means) from binary logistic models.

### Patient and public involvement

Patients (other than as participants) were not involved in the study. However, findings are being made publicly available through open access journal articles and study reports.

## Results

The sample and, for comparison, national demographics are shown in online supplemental table A2. Just over half of the sample were women (54.5%); 47.1% were aged 18–49 years and 52.9% were aged ≥50 years; 95.6% were of white ethnicity; and proportions in each deprivation quintile ranged from 19.3% (quintile 1, most deprived) to 20.6% (quintile 5, least deprived). Overall, the sample was a good match to general population characteristics for ethnicity and deprivation but included a lower percentage of individuals aged 18–29 years (14.4%) and more women (54.5%) than the national population (19.2% and 51.1%, respectively). Over half of all participants reported 0 ACEs (56.6%), with 17.9% reporting 1 ACE, 14.6% 2–3 ACEs and 10.9% ≥4 ACEs.

For each trust rating, some individuals were categorised as ‘don’t know/not applicable’. In particular, online sources such as websites, health apps and social media were not rated as frequently by older respondents and several health service sources were not rated as frequently by those of other than white ethnicities. Individuals with ACEs were more likely to be able to provide a trust rating for health advice from NHS 111 and for social services. Overall reporting levels for each question are shown in table 1 with breakdowns by demographics and ACE count in online supplemental tables A3–A6.

**Table 1:** Question response rates and percentage of individuals reporting a low trust rating for different sources of advice and services by exposure to adverse childhood experiences

	No of individuals	Response rate(%)	All(%)	ACE count (%)				
				0	1	2–3	≥4	χ^2^, P value
Low trust in health advice from								
General practitioners	1845	98.1	7.7	6.4	8.3	7.4	13.9	13.641, 0.003
Hospital doctors	1819	96.8	4.9	3.5	4.6	5.9	11.1	21.379, <0.001
Nurses	1827	97.2	3.2	2.6	1.8	3.4	8.7	22.272, <0.001
Pharmacists	1807	96.1	3.2	2.4	1.8	4.5	7.9	18.723, <0.001
NHS 111	1307	69.5	10.3	9.2	7.3	11.9	17.9	14.079, 0.003
Virtual health professionals	831	44.2	19.1	17.1	18.1	18.3	29.6	8.965, 0.030
Friends, family or colleagues	1700	90.4	15.5	13.4	16.3	19.3	19.8	8.839, 0.032
Low trust in services/systems								
Health services	1846	98.2	12.6	10.0	10.2	15.6	26.8	46.560, <0.001
Social services	1101	58.6	25.0	20.1	24.6	26.4	41.7	30.693, <0.001
Police	1705	90.7	18.8	15.0	16.5	19.4	41.3	72.730, <0.001
Charities	1580	84.0	9.4	7.7	6.8	13.9	16.0	20.131, <0.001
Government	1831	97.4	62.2	55.8	65.4	69.5	80.0	52.667, <0.001
Low trust in general advice/info from								
NHS websites	1416	75.3	7.9	6.2	5.5	12.7	13.8	19.309, <0.001
Health apps	923	49.1	34.5	32.2	35.8	29.5	49.5	13.970, 0.003
General internet sites	1517	80.7	34.4	30.7	36.8	36.5	44.9	14.817, 0.002
Social media	1414	75.2	71.5	68.1	74.5	74.8	77.5	9.701, 0.021
TV/radio programmes	1750	93.1	24.6	20.9	25.5	30.0	35.5	23.153, <0.001

ACEadverse childhood experience

### Trust in health advice

Ability to provide a trust rating in health advice was high for most types of health service staff (>96%) and friends, family or colleagues (90.4%), falling to 69.5% for NHS 111 and 44.2% for virtual health professionals (table 1). Less than 5% of those rating health advice from hospital doctors, nurses and pharmacists, and 7.7% of those rating GPs, reported low trust in this advice (table 1). Low trust increased for remote health advice delivery methods, to 1 in 10 (10.3%) of those rating NHS 111 and almost 1 in 5 (19.1%) of those rating virtual health professionals. Overall, friends, family or colleagues were less trusted than NHS 111 but more trusted than virtual health professionals (table 1).

**Figure 1:**
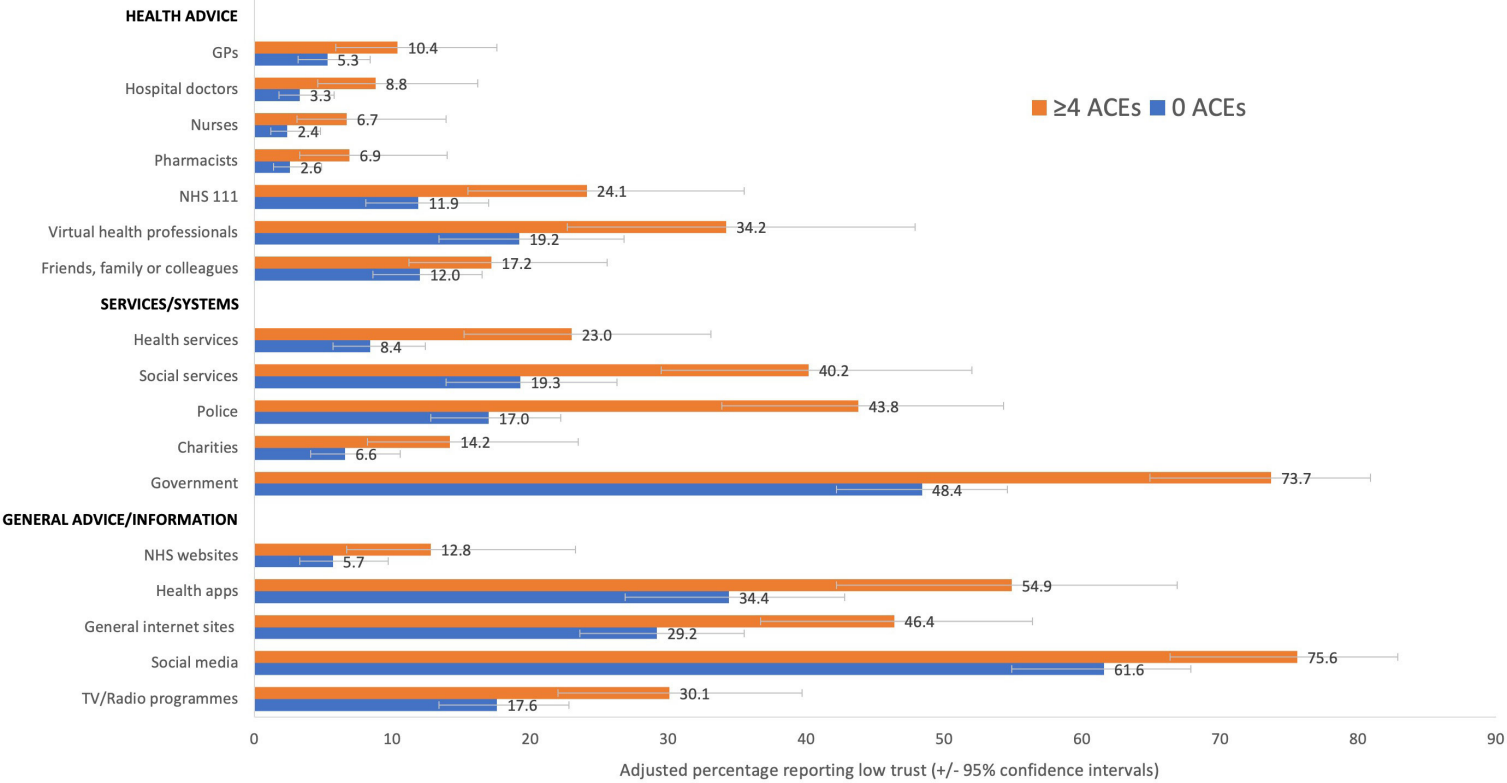
Adjusted percentages reporting low trust in different sources of advice and information, by count of adverse childhood experiences (ACE).

After accounting for sociodemographic confounders (age, sex, ethnicity and deprivation), ACE count was found to be significantly associated with low trust in health advice from each health professional source, with the likelihood of a low trust response being more than twice as high in those with ≥4 ACEs (vs 0 ACEs, table 2). There was a smaller increase in low trust in health advice from friends, family or colleagues but this was still significantly different between the 0 and ≥4 ACEs categories (table 2). Adjusted means identified an increase in low trust prevalence for health advice from hospital doctors from 3.3% (0 ACEs) to 8.8% (≥4 ACEs), and for health advice from GPs from 5.3% to 10.4%, respectively (figure 1). For health advice from NHS 111, low trust increased from 11.9% (0 ACEs) to 24.1% (≥4 ACEs) and for virtual health professionals from 19.2% to 34.2%, respectively (figure 1).

**Table 2:** Logistic regression analysis of low trust in health advice from different sources by exposure to adverse childhood experiences and sociodemographics

	General practitioners	Hospital doctors	Nurses	Pharmacists	NHS 111	Virtual health professionals	Friends, family or colleagues
	aOR (95% CI, P value)	aOR (95% CI, P value)	aOR (95% CI, P value)	aOR (95% CI, P value)	aOR (95% CI, P value)	aOR (95% CI, P value)	aOR (95% CI, P value)
ACE count							
0	Ref (0.027)	Ref (0.004)	Ref (0.004)	Ref (0.007)	Ref (0.001)	Ref (0.022)	Ref (0.058)
1	1.33 (0.84 to 2.13, 0.228)	1.27 (0.68 to 2.36, 0.458)	0.65 (0.26 to 1.60, 0.349)	0.68 (0.28 to 1.69, 0.410)	0.75 (0.43 to 1.31, 0.314)	1.10 (0.67 to 1.79, 0.711)	1.23 (0.86 to 1.76, 0.259)
2–3	1.13 (0.67 to 1.91, 0.656)	1.62 (0.87 to 2.98, 0.126)	1.20 (0.55 to 2.61, 0.643)	1.74 (0.86 to 3.55, 0.126)	1.41 (0.86 to 2.33, 0.178)	1.10 (0.66 to 1.83, 0.709)	1.55 (1.07 to 2.24, 0.020)
≥4	2.09 (1.29 to 3.40, 0.003)	2.84 (1.60 to 5.03, <0.001)	2.87 (1.50 to 5.52, 0.002)	2.74 (1.39 to 5.41, 0.004)	2.36 (1.44 to 3.89, 0.001)	2.19 (1.32 to 3.62, 0.002)	1.53 (1.00 to 2.33, 0.049)
Age (years)							
18–29	Ref (0.035)	Ref (0.026)	Ref (0.088)	Ref (0.077)	Ref (0.180)	Ref (0.001)	Ref (0.018)
30–49	1.01 (0.58 to 1.73, 0.986)	0.62 (0.33 to 1.15, 0.129)	0.71 (0.34 to 1.51, 0.376)	0.90 (0.41 to 1.96, 0.784)	0.80 (0.46 to 1.38, 0.416)	0.97 (0.57 to 1.64, 0.896)	1.16 (0.76 to 1.77, 0.504)
50–69	1.41 (0.82 to 2.42, 0.211)	1.03 (0.57 to 1.88, 0.916)	1.09 (0.52 to 2.29, 0.814)	1.31 (0.60 to 2.83, 0.497)	1.30 (0.75 to 2.26, 0.349)	2.12 (1.26 to 3.59, 0.005)	1.30 (0.85 to 2.00, 0.232)
≥70	0.64 (0.33 to 1.24, 0.189)	0.38 (0.17 to 0.88, 0.023)	0.33 (0.11 to 0.98, 0.046)	0.32 (0.10 to 1.07, 0.064)	1.17 (0.62 to 2.23, 0.628)	1.21 (0.60 to 2.45, 0.601)	0.69 (0.42 to 1.14, 0.144)
Sex*							
Women	1.19 (0.83 to 1.69, 0.340)	1.37 (0.88 to 2.14, 0.169)	1.68 (0.96 to 2.94, 0.070)	1.06 (0.62 to 1.82, 0.821)	0.70 (0.48 to 1.00, 0.051)	0.97 (0.68 to 1.39, 0.852)	0.93 (0.71 to 1.21, 0.586)
Ethnicity*							
Other than white	0.79 (0.31 to 2.03, 0.626)	0.98 (0.34 to 2.83, 0.976)	1.17 (0.35 to 3.95, 0.803)	1.60 (0.55 to 4.67, 0.390)	1.91 (0.89 to 4.10, 0.095)	1.68 (0.79 to 3.60, 0.181)	0.88 (0.44 to 1.76, 0.720)
Deprivation							
(Least) 5	Ref (0.011)	Ref (0.165)	Ref (0.249)	Ref (0.328)	Ref (0.665)	Ref (0.121)	Ref (0.011)
4	1.16 (0.66 to 2.02, 0.613)	0.96 (0.45 to 2.06, 0.921)	0.87 (0.33 to 2.30, 0.779)	1.25 (0.46 to 3.40, 0.670)	1.03 (0.59 to 1.79, 0.929)	1.07 (0.64 to 1.78, 0.798)	0.67 (0.45 to 1.00, 0.049)
3	0.56 (0.29 to 1.09, 0.086)	0.93 (0.44 to 2.00, 0.860)	0.91 (0.35 to 2.33, 0.841)	1.78 (0.70 to 4.54, 0.229)	1.12 (0.65 to 1.93, 0.692)	0.68 (0.39 to 1.18, 0.171)	0.48 (0.32 to 0.74, 0.001)
2	1.44 (0.84 to 2.48, 0.188)	1.56 (0.78 to 3.15, 0.212)	1.67 (0.71 to 3.90, 0.238)	1.55 (0.59 to 4.08, 0.373)	0.73 (0.40 to 1.34, 0.303)	0.53 (0.29 to 0.96, 0.036)	0.60 (0.40 to 0.90, 0.014)
(Most) 1	1.63 (0.96 to 2.77, 0.074)	1.79 (0.91 to 3.53, 0.094)	1.78 (0.78 to 4.09, 0.172)	2.38 (0.97 to 5.86, 0.059)	0.87 (0.49 to 1.56, 0.638)	0.75 (0.44 to 1.28, 0.295)	0.69 (0.46 to 1.03, 0.066)

P values in Rreference (Ref) rows refer to overall contribution of variable to model.

* Reference categories for sex and ethnicity awere maleen and white, respectively.

ACEadverse childhood experienceaOR, adjusted ORRef, reference category

Low trust in hospital doctors and nurses was least common in those aged ≥70 years, while low trust in virtual health professionals was highest in those aged 50–69 years (table 2). Sex and ethnicity were not independently associated with trust in any source of health advice, while low trust in GPs was higher in the more deprived quintiles and low trust in friends, family or colleagues highest in the least deprived quintile (table 2 and online supplemental table A7).

### Trust in services and systems

Nearly all (>97%) respondents provided a trust rating for government and health services, and 90.7% rated police. However, only 84.0% provided a rating for charities and 58.6% for social services (table 1). Trust was highest for charities, for which only 9.4% of respondents provided a low trust rating, followed by health services (12.6%). The prevalence of low trust was nearly twice as high for social services (25.0%) with police at 18.8%. However, low trust was substantively higher for government (62.2%) than for any other service or system.

Low trust in all services and systems increased significantly with ACE count, with those with ≥4 ACEs being over three times more likely to have low trust in health services (adjusted OR (aOR) 3.23) and police (aOR 3.81) than those with 0 ACEs (table 3). The likelihood of a low trust rating increased with even one ACE (vs 0 ACEs) for government and with two or more ACEs for health services, social services and charities. Adjusted means identified an increase in low trust in health services from 8.4% with 0 ACEs to 23.0% with ≥4 ACEs and for police from 17.0% to 43.8%, respectively (figure 1).

**Table 3:** Logistic regression analysis of low trust in different services and systems by exposure to adverse childhood experiences and sociodemographics

	Health services	Social services	Police	Charities	Government
	aOR (95% CI, P value)	aOR (95% CI, P value)	aOR (95% CI, P value)	aOR (95% CI, P value)	aOR (95% CI, P value)
ACE count					
0	Ref (<0.001)	Ref (<0.001)	Ref (<0.001)	Ref (<0.001)	Ref (<0.001)
1	1.01 (0.67 to 1.52, 0.981)	1.26 (0.86 to 1.86, 0.235)	1.08 (0.76 to 1.53, 0.688)	0.92 (0.54 to 1.58, 0.772)	1.45 (1.11 to 1.88, 0.006)
2–3	1.68 (1.13 to 2.49, 0.011)	1.50 (1.01 to 2.24, 0.046)	1.37 (0.95 to 1.98, 0.092)	2.10 (1.34 to 3.31, 0.001)	1.82 (1.36 to 2.45, <0.001)
≥4	3.23 (2.19 to 4.77, <0.001)	2.81 (1.89 to 4.18, <0.001)	3.81 (2.67 to 5.44, <0.001)	2.35 (1.44 to 3.84, 0.001)	2.99 (2.05 to 4.36, <0.001)
Age (years)					
18–29	Ref (0.001)	Ref (0.009)	Ref (<0.001)	Ref (0.076)	Ref (<0.001)
30–49	0.75 (0.48 to 1.17, 0.197)	0.93 (0.61 to 1.42, 0.737)	0.57 (0.39 to 0.83, 0.003)	0.85 (0.48 to 1.50, 0.566)	1.14 (0.83 to 1.56, 0.415)
50–69	1.42 (0.92 to 2.18, 0.110)	1.39 (0.91 to 2.12, 0.132)	1.12 (0.78 to 1.62, 0.532)	1.49 (0.86 to 2.58, 0.156)	1.60 (1.16 to 2.21, 0.004)
≥70	0.78 (0.48 to 1.30, 0.343)	0.68 (0.41 to 1.14, 0.146)	0.62 (0.40 to 0.96, 0.031)	1.25 (0.68 to 2.29, 0.479)	0.79 (0.57 to 1.10, 0.164)
Sex*					
Women	0.95 (0.72 to 1.26, 0.738)	0.75 (0.56 to 0.99, 0.041)	0.74 (0.57 to 0.95, 0.018)	0.71 (0.51 to 1.01, 0.055)	0.73 (0.60 to 0.89, 0.002)
Ethnicity*					
Other than white	0.71 (0.32 to 1.59, 0.408)	1.01 (0.49 to 2.08, 0.972)	1.39 (0.75 to 2.55, 0.294)	0.81 (0.31 to 2.08, 0.654)	0.52 (0.32 to 0.83, 0.006)
Deprivation					
(Least) 5	Ref (0.535)	Ref (0.445)	Ref (0.021)	Ref (0.051)	Ref (0.003)
4	1.05 (0.66 to 1.67, 0.842)	1.08 (0.69 to 1.68, 0.744)	1.30 (0.86 to 1.97, 0.218)	1.13 (0.63 to 2.03, 0.691)	1.38 (1.02 to 1.87, 0.035)
3	1.20 (0.76 to 1.88, 0.441)	0.78 (0.49 to 1.25, 0.304)	1.02 (0.66 to 1.56, 0.939)	1.13 (0.63 to 2.04, 0.688)	1.29 (0.96 to 1.74, 0.092)
2	1.41 (0.90 to 2.21, 0.137)	1.21 (0.78 to 1.89, 0.402)	1.52 (1.01 to 2.30, 0.045)	1.37 (0.77 to 2.43, 0.281)	1.80 (1.32 to 2.45, <0.001)
(Most) 1	1.30 (0.83 to 2.05, 0.251)	1.04 (0.66 to 1.62, 0.869)	1.75 (1.17 to 2.62, 0.007)	2.04 (1.19 to 3.51, 0.010)	1.59 (1.17 to 2.17, 0.003)

P values in reference (Ref) rows refer to overall contribution of variable to model.

* Reference categories for sex and ethnicity awere maleen and white, respectively.

ACEadverse childhood experienceaOR, adjusted ORRef, reference category

For all services and systems, adjusted odds of low trust ratings were highest among those aged 50–69 years (table 3). Men were more likely to report low trust in social services, police and government than women. Being resident in more deprived quintiles was also associated with low trust in police and government, and white ethnicity with low trust in government (table 3 and online supplemental table A7).

### Trust in general advice and information

Nearly all respondents provided a trust rating for TV/radio programmes (93.1%), 80.7% for general internet sites, and around three quarters for NHS websites and social media, although less than half (49.1%) provided a rating for health apps (table 1). Only 7.9% of those rating NHS websites were categorised as low trust, increasing to a quarter (24.6%) for TV/radio programmes and to over a third for general internet sites (34.4%) and health apps (34.5%; table 1). Trust in general advice/information from social media was lowest, with trust levels of 7 in 10 respondents (71.5%) categorised in the low category (table 1).

ACE count was independently associated with low trust responses across all sources, with the likelihood of a low trust response being around double in those with≥4 ACEs (vs 0 ACEs; table 4). For social media and general internet sites, low trust increased significantly even with one ACE (vs 0) and for NHS websites and TV/radio programmes with ≥2 ACEs. Adjusted means identified an increase in prevalence of low trust in NHS websites from 5.7% with 0 ACEs to 12.8% with ≥4 ACEs and for TV/radio programmes from 17.6% to 30.1%, respectively (figure 1).

**Table 4:** Logistic regression analysis of low trust in different sources of general advice and information by exposure to adverse childhood experiences and sociodemographics

	NHS websites	Health apps	General internet sites	Social media	TV/radio programmes
	aOR (95% CI, P value)	aOR (95% CI, P value)	aOR (95% CI, P value)	aOR (95% CI, P value)	aOR (95% CI, P value)
ACE count					
0	Ref (0.001)	Ref (0.001)	Ref (<0.001)	Ref (0.001)	Ref (<0.001)
1	0.88 (0.48 to 1.62, 0.684)	1.21 (0.83 to 1.75, 0.322)	1.37 (1.03 to 1.83, 0.031)	1.48 (1.07 to 2.04, 0.018)	1.27 (0.94 to 1.71, 0.121)
2–3	2.19 (1.32 to 3.64, 0.003)	0.92 (0.62 to 1.36, 0.664)	1.41 (1.04 to 1.92, 0.028)	1.53 (1.09 to 2.16, 0.015)	1.60 (1.17 to 2.19, 0.003)
≥4	2.45 (1.38 to 4.33, 0.002)	2.32 (1.50 to 3.59, <0.001)	2.10 (1.49 to 2.96, <0.001)	1.93 (1.29 to 2.90, 0.002)	2.02 (1.43 to 2.86, <0.001)
Age (years)					
18–29	Ref (0.007)	Ref (0.001)	Ref (0.001)	Ref (<0.001)	Ref (0.018)
30–49	0.91 (0.47 to 1.73, 0.765)	1.25 (0.83 to 1.88, 0.297)	1.19 (0.86 to 1.65, 0.294)	1.65 (1.21 to 2.26, 0.002)	1.27 (0.89 to 1.82, 0.187)
50–69	1.98 (1.07 to 3.67, 0.031)	2.13 (1.40 to 3.26, <0.001)	1.75 (1.25 to 2.44, 0.001)	2.22 (1.57 to 3.15, <0.001)	1.60 (1.12 to 2.30, 0.010)
≥70	1.70 (0.80 to 3.60, 0.170)	1.81 (1.05 to 3.11, 0.034)	1.77 (1.18 to 2.63, 0.005)	1.86 (1.19 to 2.92, 0.007)	1.06 (0.71 to 1.59, 0.768)
Sex*					
Women	1.12 (0.75 to 1.69, 0.573)	0.94 (0.71 to 1.25, 0.669)	0.88 (0.71 to 1.10, 0.266)	0.93 (0.73 to 1.18, 0.561)	0.90 (0.72 to 1.12, 0.351)
Ethnicity*					
Other than white	0.97 (0.34 to 2.82, 0.961)	1.36 (0.71 to 2.59, 0.352)	0.92 (0.54 to 1.57, 0.766)	0.59 (0.36 to 0.97, 0.038)	0.70 (0.38 to 1.28, 0.247)
Deprivation					
(Least) 5	Ref (0.005)	Ref (0.483)	Ref (0.749)	Ref (0.031)	Ref (0.041)
4	2.60 (1.37 to 4.92, 0.003)	1.29 (0.85 to 1.96, 0.235)	0.86 (0.62 to 1.20, 0.377)	0.69 (0.47 to 1.02, 0.062)	1.51 (1.05 to 2.16, 0.026)
3	1.35 (0.67 to 2.74, 0.401)	1.09 (0.71 to 1.68, 0.698)	0.81 (0.58 to 1.13, 0.211)	0.60 (0.41 to 0.87, 0.007)	1.74 (1.22 to 2.48, 0.002)
2	1.23 (0.59 to 2.59, 0.582)	0.87 (0.55 to 1.37, 0.549)	0.84 (0.60 to 1.18, 0.319)	0.75 (0.51 to 1.11, 0.151)	1.37 (0.95 to 1.99, 0.092)
(Most) 1	2.50 (1.29 to 4.85, 0.006)	0.96 (0.62 to 1.50, 0.862)	0.84 (0.59 to 1.18, 0.305)	0.56 (0.38 to 0.83, 0.004)	1.53 (1.06 to 2.21, 0.024)

P values in reference (Ref) rows refer to overall contribution of variable to model.

* Reference categories for sex and ethnicity weare maleen and white, respectively.

ACEadverse childhood experienceaOR, adjusted ORRef, reference category

For all sources, higher proportions of low trust were reported by those aged ≥50 years, with the exception of trust in TV/radio programmes and NHS websites by those aged ≥70 years (table 4). Low trust did not vary significantly with sex or ethnicity, except for a lower likelihood of low trust in social media in other than white ethnicities. Low trust in NHS websites was highest in deprivation quintiles 1 (most deprived) and 4. Low trust in social media was most likely in deprivation quintile 5 (least deprived), while low trust in TV/radio programmes was least likely in this quintile (table 4 and online supplemental table A7).

## Discussion

Positive relationships with parents and other caregivers in childhood are related to stronger attachments,^39 40^ and help develop the ability to trust others.^41^ When children have been hurt or neglected by caregivers, their ability to trust others may be diminished along with their ability to form attachment bonds,^42^,^44^ even subsequently with their own children.^45^ Moreover, maltreated children can be more likely to interpret neutral facial expressions as angry or aggressive,^46^ with immediate implications for developing attachment and trust.^43^ While such limited findings illustrate the impacts ACEs may have on an individual’s ability to trust others, less empirical information is available on how ACEs may affect trust in information and advice from professionals, and public and private organisations. In a largely separate literature, trust in organisations often considers issues such as their consistency, reliability, transparency, ethics and integrity, and proven competence.^47^,^50^ However, such work has generally not considered how individuals’ experiences of ACEs may affect their ability to trust information and advice from organisations and professionals for health and other purposes.

Here, our results identified strong associations between exposure to ACEs and greater prevalence of low trust in different support systems, as well as health and other advice and information from professionals, agencies and information sources. Of particular concern to healthcare and public health systems, individuals with ≥4 ACEs (vs 0 ACEs) were between two and three times more likely to have low trust in GPs, hospital doctors, nurses and pharmacists (table 2). Critically, those with a high ACEs count are more likely to have greater healthcare, health protection and health promotion needs.^6^ Consequently, low trust may result in poorer uptake of health improving behaviour advice, specifically in those most likely to develop non-communicable diseases, infections and other physical and mental health issues at earlier ages. Furthermore, trust in sources of advice is an important predictor of compliance with such advice.^51^ Consequently, individuals with higher ACE counts may also be less likely to adopt and maintain fidelity to medication regimens, other treatment plans or vaccination schedules.^26 52^ Health systems should recognise that those most in need of their support may also be least likely to trust their advice. Recent developments in trauma informed approaches to health and well being have begun considering how all systems may better meet the needs of those who have experienced trauma, including ACEs.^53 54^ However, further empirical work is urgently needed, especially when exposure to ACEs remains a common experience (here, 43.4% had at least one ACE and 10.9% ≥4 ACEs).

Overall, low trust was much more prevalent in advice and information from virtual (online and apps) or remote (NHS 111) health advice sources than directly from health professionals (table 1), although response levels varied by source (see limitations). Sources carrying the NHS brand or associated with health professions were more trusted than their more generic counterparts (eg, low trust in NHS websites vs general internet sites, table 1), with social media the least trusted source (71.5% low trust; table 1). Regardless, there were strong positive associations between multiple ACE exposure and low trust for all such sources (tables2^4^). With increasing proportions of public health advice and service contact moving to remote platforms (eg, phone and internet), lower trust in these services should be of concern. Moreover, individuals with higher ACE counts are often greater users of health systems and, for instance, adjusted prevalence of low trust in health advice from the NHS 111 service rose from 11.9% with 0 ACEs to 24.1% with ≥4 ACEs (figure 1).

Similar increases in low trust with higher ACEs were seen for all services, including health services (tables1  3). The greatest increase in the likelihood of low trust with ACEs was seen for police (≥4 ACEs vs 0 ACEs; aOR 3.81), although adjusted odds also at least doubled in those with ≥4 ACEs for health and social services, charities and government (table 3). While evidence supports individuals with higher ACEs having more experiences as perpetrators and victims of crime,^7 55^ results here identified an estimated increase in prevalence of low trust in police services from 17.0% (0 ACEs) to 43.8% (≥4 ACEs; figure 1). Some police forces are already considering how they make services more trauma informed in order to better engage and build trust with those with a history of adversity.^56 57^ Typically, however, in the UK and elsewhere, such measures are not yet an integrated feature of police training and operations.

Across all services and systems, the highest prevalence of reported low trust was seen for government (table 1). However, public health advice is frequently referred to as government guidance or guidelines and issued by government departments. Examples include advice for healthy eating^58^ and physical activity,^59^ while in some countries health warning labels on alcohol products are identified as governmental rather than health service or medical advice (eg, USA;^60^). Our results suggested labelling information and advice as governmental may lead to lower levels of trust in it and potentially provide commercial organisations wishing to lessen its uptake an opportunity to exploit a less trusted title (eg, governmental guidance). The differences in trust for those with high levels of ACEs were particularly stark. An estimated 23.0% of those with ≥4 ACEs reported low trust in health services, rising to 73.7% with low trust in government (figure 1). Consequently, our results suggest that provision of national health related or other guidance should consider the benefits of an exclusive health service brand rather than a governmental one.

Finally, our results suggested very low levels of trust in social media, with considerably greater trust in more traditional information platforms, such as TV/radio programmes (table 1). Again, for all communication platforms, low trust was significantly increased in those with higher ACEs (table 4, figure 1). However, increasing amounts of public funding are being invested in social media messaging in order to reach different population groups. The value of such investments may need recalibrating if three quarters of those with high ACE counts have low trust in social media and even among those with no ACE exposure most individuals have low trust in advice and information on such platforms.

### Limitations

The study relied on recall of ACEs by adults, which may be incomplete or inaccurate and, in some cases, participants may have chosen not to disclose certain ACEs despite the reassurance of confidentiality and anonymity. Moreover, an average interview time of 22 min may have impacted some respondents’ focus and compliance over the course of their interview. However, ACE prevalence was comparable with other studies undertaken in the UK.^35^ While compliance levels were also generally consistent with other ACE surveys (49%^35^) we cannot identify how ACEs and levels of trust in those choosing not to complete the survey may have affected the results. Here, respondents were able to say if they could not rate trust in any particular individual, agency or service (table 1; online supplemental tables A3–A6) as not all respondents may feel adequately exposed to or informed on each source. The majority of respondents (>90%) could rate each type of individual health professional (ie, GPs, hospital doctors, nurses and pharmacists) and there were no significant relationships between ACE counts and providing such trust ratings (online supplemental table A4). For some information/advice sources and services, ACE count was related to ability to provide a trust rating. Thus social services and NHS 111 were more likely to be given a rating by individuals with higher ACEs (online supplemental tables A4 and A5), which may reflect a greater likelihood of being in contact with such services. Individuals with 2–3 ACEs (vs 0 ACEs) were more likely to rate health apps, general internet sites, virtual health professionals and social media but less likely to rate health services in general (online supplemental tables A4–A6). It is not possible to assess whether this reflects a pattern of accessing remote services more among this group. However, results may better reflect those directly or indirectly exposed to each source rather than any overall population wide prevalence.

We used a range of ACEs typically identified in ACE research tools used by the World Health Organization and US Centers for Disease Control and Prevention. However, there is continuing debate about whether other childhood adversities (eg, peer victimisation, community violence) should be included in ACE measurements^61^,^63^ and how this would affect relationships with trust requires further studies. Moreover, our results did not measure the severity of ACEs, length of exposure, frequency and age of occurrence,^61^ all of which may be important in the development of trust. With little work having already been undertaken on the impacts of adversity on trust in the range of individuals and organisations we examined, there were no validated instruments, and the questions used here require further validation and refinement. Our questions did not examine if levels of trust might vary depending on the type of information or service provided but this is an important consideration for further studies. We chose to dichotomise participants trust ratings into low trust (yes 0–4, or no 5–10 on the scale) with the same single cut-off applied to all items. This reflected the aim of the study to examine factors relating specifically to low trust. However, further studies could examine other categorisations including, for instance, very high or very low trust (eg, 8–10 or 0–2 on the 0–10 scale, respectively). Finally, the survey was carried out after a pandemic and during a cost of living crisis,^64^ and we were not able to ascertain whether trust in information and advice from different sources was affected by these global events and whether any such affects are sustained.

## Conclusions

In the UK and elsewhere, trust in institutions and figures of professional standing or other authority has diminished over recent decades.^65^ However, public health and healthcare systems rely on trust as a mechanism to ensure advice is followed and for fidelity to treatment. The alternative, seen during the COVID-19 pandemic, is the use of legislation and criminal justice agencies to enforce health measures (eg, restricting movements). The choice of institution or professional with which information is associated may be a critical decision in its credibility. Individuals with ACEs are an important population for health, social, criminal justice and other sectors to influence. Those with ACEs may require disproportionate amounts of public sector or charitable support, and may benefit more from services but be less likely to trust in such services and the advice they provide. Greater trust in services by those with healthier childhoods will only serve to perpetuate intergenerational cycles of adversity and associated inequalities. Thus trauma informed approaches are required that understand who the most credible communicators are and how best to develop trust in essential support services.

## supplementary material

10.1136/bmjph-2023-000868 online supplemental file 1

## Data Availability

Data are available upon reasonable request.
