# Supplementary material for: Associations between adverse childhood experiences and trust in health and other information from public services, professionals and wider sources: national cross sectional survey
Source: BMJ Public Health. 2024 May 27;2(1):e000868. doi: 10.1136/bmjph-2023-000868 (PMC11812900; doi:10.1136/bmjph-2023-000868)
Supplement: online supplemental file 1 [file bmjph-2-1-s001.pdf]

Table A1. Questions and qualifying responses

	Question *(response options)*	Qualifying response
**ACEs**	All ACE questions were preceded by the statement "While you were growing up, before the age of 18 years..."	
Physical abuse	How often did a parent or adult in your home ever hit, beat, kick, or physically hurt you in any way? This does not include gentle smacking for punishment *(never; once; more than once; prefer not to say)*	Once or more than once
Verbal abuse	How often did a parent or adult in your home ever swear at you, insult you, or put you down? *(never; once; more than once; prefer not to say)*	More than once
Sexual abuse	Did an adult or someone at least five years older than you sexually abuse you by touching you or making you undertake any sexual activity with them? *(no; yes; prefer not to say)*	Yes
Parental separation	Were your parents ever separated or divorced? *(no; yes; prefer not to say)*	Yes
Domestic violence	How often did your parents or adults in your home ever slap, kick, punch, or beat each other up? *(never; once; more than once; prefer not to say)*	Once or more than once
Mental illness	Did you live with anyone who was depressed, mentally ill or suicidal? *(no; yes; prefer not to say)*	Yes
Alcohol abuse	Did you live with anyone who was a problem drinker or alcoholic? *(no; yes; prefer not to say)*	Yes
Drug abuse	Did you live with anyone who used illegal street drugs or abused prescription medications? *(no; yes; prefer not to say)*	Yes
Incarceration	Did you live with anyone who served time or was sentenced to serve time in a prison or young offenders' institution? *(no; yes; prefer not to say)*	Yes
**Use and perceptions of health and other services variables**		
Low trust in health and other services	On a scale of 0 to 10, where 0 is not at all and 10 is completely, how much do you trust the following…? *(0, not at all - 10, completely; don't know/not applicable)*	0-4
	Health services	
	Social services	
	Police	
	Charities/voluntary organisations	
	Government	
Low trust in general advice and information sources	On a scale of 0 to 10, where 0 is not at all and 10 is completely, how much do you trust general advice and information from…? *(0, not at all - 10, completely; don't know/not applicable)*	0-4
	TV/radio programmes	
	NHS websites	
	Other internet sites/internet searches (e.g. Google, YouTube, Wikipedia)	
	Social media such as Twitter and Facebook	
	Health apps for smartphones or tablets	
Low trust in health advice	On a scale of 0 to 10, where 0 is not at all and 10 is completely, how much would you trust health advice given to you by the following individuals…? *(0, not at all - 10, completely; don't know/not applicable)*	0-4
	GPs (general practitioners)	
	Hospital doctors	
	Nurses	
	Pharmacists/chemists	
	Friends, family or colleagues	
	Health professionals available through NHS 111	
	Health professionals accessed via other means e.g. an app*	

ACE = Adverse childhood experiences. TV = Television. NHS = National Health Service. App = Application. *Termed in text as *virtual health professionals*.

Table A2: Sample demographics

	Sample		Welsh population*
	n	%	%
**ACE count**			
0	1065	56.6	
1	337	17.9	
2-3	274	14.6	
≥4	204	10.9	
**Age (years)**			
18-29	271	14.4	19.2
30-49	614	32.7	29.4
50-69	564	30.0	32.2
70+	431	22.9	19.2
**Sex**			
Male	856	45.5	48.9
Female	1024	54.5	51.1
**Ethnicity**			
White	1797	95.6	95.2
Other than white	83	4.4	4.8
**Deprivation**			
(Least) 5	387	20.6	18.7
4	379	20.2	19.5
3	385	20.5	20.9
2	367	19.5	20.8
(Most) 1	362	19.3	20.2

*Sources

Age, sex and deprivation: Office for National Statistics. Mid-2020 (30 June) estimates of the usual resident population for Lower layer Super Output Areas (LSOAs) in England and Wales by single year of age and sex. https://www.ons.gov.uk/peoplepopulationandcommunity/populationandmigration/populationestimates/datasets/lowersuperoutputareamidyearpopulationestimates

Ethnicity: StatsWales. Ethnicity by area and ethnic group (year ending 31 March 2023). https://statswales.gov.wales/Catalogue/Equality-and-Diversity/Ethnicity/ethnicity-by-area-ethnicgroup

Table A3. Percentage of individuals providing a trust rating for different sources of advice and services by exposure to adverse childhood experiences and socio-demographics

	ACE count						Age (years)						Sex				Ethnicity				Deprivation						
	0	1	2-3	≥4	X^2	P	18-29	30-49	50-69	70+	X^2	P	Male	Female	X^2	P	White	Other than white	X^2	P	5 (least)	4	3	2	1 (most)	X^2	P
**n**	1065	337	274	204			271	614	564	431			856	1024			1797	83			*387*	*379*	*385*	*367*	*362*		
**Low trust in:**																											
**health advice from:**																											
GPs	97.5	99.7	98.2	99.0	8.03	0.045	96.7	98.7	98.2	98.1	4.23	0.237	97.9	98.3	0.50	0.479	98.3	94.0	8.23	0.004	97.9	98.9	97.7	98.6	97.5	3.19	0.527
Hospital doctors	96.3	96.7	98.2	97.1	2.41	0.492	97.0	96.9	97.7	95.1	5.34	0.148	96.5	97.0	0.34	0.561	97.1	90.4	11.31	<0.001	97.4	96.3	95.6	97.3	97.2	3.05	0.550
Nurses	97.2	98.8	96.4	95.6	5.86	0.119	96.3	97.2	97.7	97.0	1.36	0.715	96.5	97.8	2.70	0.101	97.6	89.2	20.41	<0.001	96.9	95.8	97.4	98.6	97.2	5.75	0.219
Pharmacists	96.2	97.0	96.4	93.6	4.23	0.238	91.9	96.7	98.0	95.4	19.97	<0.001	94.6	97.4	9.36	0.002	96.4	89.2	11.27	<0.001	97.4	95.5	94.5	97.8	95.3	8.16	0.086
NHS 111	64.6	73.3	76.6	79.4	30.41	<0.001	73.4	82.2	68.8	49.9	127.47	<0.001	66.8	71.8	5.40	0.020	69.5	69.9	0.01	0.942	69.0	68.6	69.9	70.3	69.9	0.35	0.986
Virtual health professionals	40.0	46.0	51.8	52.9	20.84	<0.001	57.9	53.9	44.3	21.6	133.63	<0.001	42.2	45.9	2.62	0.105	43.9	50.6	1.44	0.230	47.5	42.7	41.6	40.9	48.3	7.34	0.119
Friends, family or colleagues	89.3	92.9	92.7	89.2	5.90	0.117	86.0	92.7	92.0	87.9	14.51	0.002	89.5	91.2	1.60	0.206	90.5	88.0	0.61	0.433	93.3	87.3	91.4	90.7	89.2	8.92	0.063
**services/systems:**																											
Health services	98.8	99.1	95.6	97.1	15.35	0.002	93.0	98.7	99.6	98.8	49.92	<0.001	97.2	99.0	8.77	0.003	98.4	94.0	8.69	0.003	98.7	97.6	98.2	98.1	98.3	1.33	0.856
Social services	53.3	59.1	65.0	76.5	43.62	<0.001	62.7	61.2	61.5	48.3	24.64	<0.001	58.4	58.7	0.02	0.902	58.9	51.8	1.63	0.201	54.0	60.4	55.6	59.7	63.5	9.14	0.058
Police	89.1	93.5	92.0	92.6	7.70	0.053	93.4	92.8	90.6	86.1	16.49	<0.001	92.4	89.3	5.48	0.019	91.2	79.5	12.84	<0.001	88.9	89.7	90.9	91.3	92.8	4.03	0.402
Charities	83.0	82.5	86.5	88.7	6.03	0.110	79.7	85.0	86.0	82.8	6.31	0.098	82.4	85.5	3.32	0.069	84.3	79.5	1.33	0.250	82.9	82.8	82.1	87.5	85.1	5.36	0.253
Government	97.0	98.5	97.1	98.0	2.78	0.427	93.7	98.0	97.7	98.4	17.22	<0.001	97.2	97.6	0.24	0.623	97.6	94.0	4.00	0.046	97.2	97.6	98.2	96.7	97.2	1.78	0.776
**general advice/information from:**																											
NHS websites	72.3	81.0	80.7	74.5	15.36	0.002	82.3	87.5	79.8	47.8	237.45	<0.001	73.6	76.8	2.50	0.114	75.4	73.5	0.16	0.693	80.1	74.7	76.1	73.3	72.1	7.80	0.099
Health apps	44.0	53.1	60.6	53.4	29.09	<0.001	62.7	59.6	50.9	23.2	163.67	<0.001	46.0	51.7	5.92	0.015	48.9	54.2	0.91	0.340	51.7	52.0	48.3	45.5	47.8	4.53	0.339
General internet sites	76.5	84.6	88.0	86.3	28.48	<0.001	94.8	94.6	83.5	48.3	405.14	<0.001	81.3	80.2	0.38	0.536	80.5	85.5	1.31	0.252	82.9	80.7	83.4	79.9	78.2	6.31	0.117
Social media	70.6	78.0	83.9	82.8	31.12	<0.001	95.9	94.5	74.6	35.5	549.22	<0.001	73.7	76.5	1.89	0.169	74.7	86.7	6.20	0.013	75.2	73.4	77.1	76.8	73.5	2.58	0.631
TV/radio programmes	92.9	94.4	93.8	91.2	2.30	0.512	93.4	94.6	94.9	88.4	19.75	<0.001	93.3	92.9	0.16	0.689	93.4	86.7	5.42	0.020	92.2	94.5	92.5	94.6	91.7	4.04	0.400

ACE = Adverse childhood experience.

Table A4. Logistic regression analysis of providing any trust rating in health advice from different sources by exposure to adverse childhood experiences and socio-demographics

	GPs			Hospital doctors			Nurses			Pharmacists			NHS 111			Virtual health professionals			Friends, family or colleagues		
	**AOR**	**95% CIs**	**P**	**AOR**	**95% CIs**	**P**	**AOR**	**95% CIs**	**P**	**AOR**	**95% CIs**	**P**	**AOR**	**95% CIs**	**P**	**AOR**	**95% CIs**	**P**	**AOR**	**95% CIs**	**P**
**ACE count**																					
0	Ref		0.086	Ref		0.598	Ref		0.088	Ref		0.160	Ref		0.003	Ref		0.084	Ref		0.098
1	9.64	1.29-71.85	0.027	1.09	0.55-2.18	0.803	2.50	0.86-7.21	0.091	1.38	0.67-2.85	0.376	1.36	1.03-1.81	0.031	1.13	0.87-1.46	0.349	1.59	1.00-2.53	0.050
2-3	1.40	0.52-3.72	0.505	1.94	0.75-5.02	0.172	0.75	0.35-1.58	0.444	1.06	0.51-2.19	0.877	1.59	1.16-2.19	0.004	1.41	1.07-1.86	0.016	1.58	0.95-2.61	0.075
≥4	2.83	0.65-12.30	0.166	1.04	0.42-2.56	0.929	0.55	0.25-1.21	0.138	0.53	0.27-1.06	0.071	1.61	1.11-2.35	0.013	1.24	0.91-1.70	0.172	1.00	0.60-1.65	0.993
**Age (years)**																					
18-29	Ref		0.216	Ref		0.113	Ref		0.728	Ref		0.001	Ref		<0.001	Ref		<0.001	Ref		0.003
30-49	2.82	1.06-7.52	0.038	0.93	0.40-2.18	0.876	1.35	0.60-3.03	0.471	2.49	1.32-4.70	0.005	1.73	1.22-2.43	0.002	0.85	0.64-1.14	0.292	2.10	1.32-3.34	0.002
50-69	1.87	0.73-4.79	0.191	1.16	0.46-2.88	0.757	1.42	0.60-3.34	0.421	3.95	1.86-8.38	<0.001	0.82	0.59-1.15	0.249	0.58	0.43-0.78	<0.001	1.89	1.18-3.02	0.008
70+	1.96	0.71-5.38	0.192	0.51	0.22-1.23	0.134	0.99	0.41-2.39	0.984	1.48	0.76-2.87	0.247	0.39	0.28-0.55	<0.001	0.20	0.14-0.29	<0.001	1.22	0.76-1.94	0.414
**Sex***																					
Female	1.18	0.60-2.33	0.627	1.10	0.66-1.84	0.714	1.59	0.91-2.78	0.104	2.08	1.27-3.41	0.003	1.19	0.97-1.46	0.100	1.12	0.92-1.36	0.251	1.18	0.86-1.61	0.302
**Ethnicity***																					
Other than white	0.28	0.10-0.79	0.016	0.25	0.11-0.57	0.001	0.18	0.08-0.40	<0.001	0.33	0.15-0.72	0.005	0.73	0.44-1.20	0.211	0.97	0.62-1.53	0.902	0.77	0.38-1.54	0.457
**Deprivation**																					
(Least) 5	Ref		0.638	Ref		0.509	Ref		0.176	Ref		0.131	Ref		0.958	Ref		0.021	Ref		0.053
4	1.94	0.58-6.55	0.285	0.66	0.29-1.51	0.322	0.78	0.36-1.68	0.520	0.59	0.26-1.31	0.193	0.89	0.65-1.22	0.473	0.74	0.55-0.99	0.045	0.48	0.29-0.79	0.004
3	0.90	0.34-2.40	0.838	0.57	0.26-1.28	0.173	1.33	0.56-3.16	0.517	0.49	0.23-1.07	0.073	0.94	0.68-1.29	0.691	0.68	0.51-0.92	0.012	0.75	0.44-1.29	0.297
2	1.51	0.49-4.71	0.476	0.94	0.38-2.30	0.887	2.58	0.89-7.48	0.082	1.31	0.50-3.39	0.583	0.92	0.66-1.27	0.611	0.64	0.48-0.87	0.004	0.69	0.40-1.18	0.173
(Most) 1	0.89	0.33-2.39	0.813	0.98	0.40-2.42	0.962	1.46	0.61-3.53	0.397	0.66	0.29-1.50	0.320	0.90	0.65-1.25	0.519	0.89	0.65-1.20	0.436	0.60	0.35-1.01	0.056

AOR = Adjusted odds ratio. ACE = Adverse childhood Experience. Cis = Confidence intervals. Ref = Reference category. *Reference category for sex and ethnicity are male and white respectively. P values in Ref rows refer to overall contribution of variable to model.

Table A5. Logistic regression analysis of providing any trust rating in different services and systems advice by exposure to adverse childhood experiences and socio-demographics

	Health services			Social services			Police			Charities			Government		
	**AOR**	**95% Cis**	**P**	**AOR**	**95% Cis**	**P**	**AOR**	**95% Cis**	**P**	**AOR**	**95% Cis**	**P**	**AOR**	**95% Cis**	**P**
**ACE count**															
0	Ref		0.008	Ref		<0.001	Ref		0.214	Ref		0.126	Ref		0.241
1	1.81	0.50-6.59	0.368	1.21	0.94-1.56	0.137	1.62	1.00-2.62	0.050	0.99	0.72-1.38	0.965	2.42	0.92-6.33	0.073
2-3	0.30	0.13-0.69	0.005	1.54	1.16-2.04	0.003	1.30	0.80-2.11	0.292	1.34	0.91-1.97	0.139	1.15	0.52-2.57	0.729
≥4	0.44	0.16-1.25	0.125	2.51	1.77-3.58	<0.001	1.24	0.69-2.21	0.471	1.61	1.01-2.59	0.047	1.95	0.67-5.74	0.223
**Age (years)**															
18-29	Ref		<0.001	Ref		0.001	Ref		0.001	Ref		0.089	Ref		0.001
30-49	5.54	2.34-13.14	<0.001	0.99	0.73-1.33	0.922	0.99	0.55-1.75	0.960	1.45	1.00-2.11	0.051	3.64	1.70-7.80	0.001
50-69	17.64	4.03-77.12	<0.001	1.02	0.75-1.39	0.879	0.66	0.37-1.17	0.153	1.63	1.11-2.40	0.013	2.92	1.37-6.20	0.005
70+	4.81	1.70-13.59	0.003	0.64	0.47-0.88	0.007	0.43	0.24-0.76	0.004	1.33	0.89-1.98	0.168	4.51	1.79-11.35	0.001
**Sex***															
Female	3.05	1.42-6.56	0.004	0.96	0.80-1.16	0.696	0.65	0.47-0.90	0.009	1.21	0.94-1.55	0.138	1.10	0.62-1.95	0.752
**Ethnicity***															
Other than white	0.27	0.09-0.78	0.015	0.68	0.43-1.07	0.095	0.26	0.15-0.47	<0.001	0.76	0.44-1.34	0.349	0.45	0.17-1.23	0.121
**Deprivation**															
(Least) 5	Ref		0.848	Ref		0.192	Ref		0.391	Ref		0.272	Ref		0.724
4	0.62	0.20-1.94	0.410	1.23	0.92-1.65	0.164	1.03	0.65-1.65	0.891	0.98	0.67-1.43	0.905	1.24	0.50-3.05	0.640
3	0.89	0.27-2.94	0.845	1.01	0.75-1.34	0.962	1.21	0.75-1.95	0.441	0.95	0.65-1.38	0.775	1.75	0.66-4.61	0.260
2	0.78	0.23-2.58	0.678	1.20	0.89-1.61	0.228	1.25	0.77-2.05	0.365	1.44	0.95-2.16	0.084	0.91	0.39-2.10	0.818
(Most) 1	1.11	0.32-3.90	0.867	1.35	1.00-1.83	0.047	1.63	0.97-2.75	0.066	1.14	0.77-1.70	0.507	1.15	0.47-2.81	0.757

AOR = Adjusted odds ratio. ACE = Adverse childhood Experience. CIs = Confidence intervals. Ref = Reference category. *Reference category for sex and ethnicity are male and white respectively. P values in Ref rows refer to overall contribution of variable to model.

Table A6. Logistic regression analysis of providing any trust rating in different sources of general advice and information by exposure to adverse childhood experiences and socio-demographics

	NHS websites			Health apps			General internet sites			Social media			TV/radio programmes		
	AOR	95% CIs	P	AOR	95% CIs	P	AOR	95% CIs	P	AOR	95% CIs	P	AOR	95% CIs	P
**ACE count**															
0	Ref		0.010	Ref		0.003	Ref		0.046	Ref		0.052	Ref		0.341
1	1.37	0.99-1.90	0.057	1.25	0.97-1.62	0.088	1.25	0.87-1.82	0.231	1.07	0.76-1.51	0.689	1.16	0.68-1.96	0.590
2-3	1.31	0.92-1.87	0.132	1.68	1.27-2.24	<0.001	1.80	1.16-2.78	0.008	1.73	1.15-2.60	0.008	1.02	0.59-1.77	0.946
≥4	0.69	0.48-1.01	0.055	1.06	0.77-1.45	0.718	0.96	0.59-1.56	0.860	0.89	0.57-1.41	0.629	0.640	0.36-1.12	0.115
**Age (years)**															
18-29	Ref		0.001	Ref		<0.001	Ref		<0.001	Ref		<0.001	Ref		<0.001
30-49	1.49	1.00-2.21	0.051	0.88	0.65-1.19	0.403	0.98	0.51-1.87	0.948	0.72	0.36-1.45	0.361	1.22	0.67-2.23	0.509
50-69	0.77	0.53-1.13	0.189	0.61	0.45-0.83	0.001	0.26	0.14-0.46	<0.001	0.12	0.06-0.23	<0.001	1.19	0.64-2.20	0.590
70+	0.17	0.12-0.25	0.001	0.18	0.13-0.25	<0.001	0.04	0.02-0.08	<0.001	0.02	0.01-0.04	<0.001	0.46	0.25-0.83	0.010
**Sex***															
Female	1.13	0.90-1.42	0.276	1.22	1.01-1.48	0.045	0.81	0.62-1.05	0.111	1.07	0.83-1.37	0.621	0.91	0.63-1.30	0.596
**Ethnicity***															
Other than white	0.54	0.32-0.93	0.025	0.92	0.58-1.46	0.736	0.56	0.28-1.12	0.101	0.80	0.38-1.68	0.554	0.38	0.19-0.75	0.006
**Deprivation**															
(Least) 5	Ref		0.005	Ref		0.037	Ref		0.002	Ref		0.114	Ref		0.509
4	0.62	0.43-0.90	0.012	0.91	0.67-1.23	0.529	0.68	0.44-1.02	0.065	0.70	0.47-1.03	0.068	1.40	0.78-2.52	0.254
3	0.66	0.45-0.95	0.026	0.75	0.56-1.02	0.062	0.80	0.52-1.22	0.293	0.86	0.58-1.27	0.439	1.01	0.59-1.73	0.977
2	0.53	0.37-0.77	0.001	0.65	0.48-0.88	0.005	0.47	0.31-0.72	<0.001	0.75	0.50-1.12	0.160	1.43	0.79-2.58	0.240
(Most) 1	0.53	0.36-0.76	0.001	0.72	0.53-0.98	0.039	0.50	0.33-0.75	0.001	0.60	0.40-0.89	0.012	0.970	0.56-1.66	0.904

AOR = Adjusted odds ratio. ACE = Adverse childhood Experience. Cis = Confidence intervals. Ref = Reference category. *Reference category for sex and ethnicity are male and white respectively. P values in Ref rows refer to overall contribution of variable to model.

Table A7. Percentage of individuals reporting a low trust rating for different sources of advice and services by socio-demographics

	Age (years)						Sex				Ethnicity				Deprivation						
**Low trust in:**	**18-29**	**30-49**	**50-69**	**70+**	**X^2**	**P**	**Male**	**Female**	**X^2**	**P**	**White**	**Other than white**	**X^2**	**P**	**5 (Least)**	**4**	**3**	**2**	**1 (Most)**	**X^2**	**P**
**health advice from:**																					
GPs	8.0	7.8	9.9	4.5	10.04	0.018	6.9	8.3	1.30	0.254	7.8	6.4	0.19	0.663	6.6	7.7	4.0	9.4	11.0	14.97	0.005
Hospital doctors	7.2	4.4	6.4	2.2	12.36	0.006	4.0	5.6	2.62	0.106	4.9	5.3	0.03	0.857	3.7	3.8	3.8	5.9	7.4	8.40	0.078
Nurses	4.6	3.2	4.2	1.2	8.67	0.034	2.3	4.0	4.16	0.041	3.2	4.1	0.17	0.682	2.4	2.2	2.4	4.1	5.1	7.84	0.098
Pharmacists	4.0	3.4	4.3	1.0	9.46	0.024	3.0	3.4	0.29	0.592	3.1	5.4	1.20	0.274	1.9	2.5	3.6	3.1	5.2	7.49	0.112
NHS 111	11.1	8.3	12.4	10.7	4.10	0.251	12.1	9.0	3.30	0.069	10.1	15.5	1.76	0.184	10.5	11.2	11.9	7.8	10.3	2.76	0.598
Virtual health professionals	15.9	14.8	27.6	17.2	16.86	0.001	19.4	18.9	0.03	0.869	18.9	23.8	0.63	0.429	22.8	23.5	16.9	12.7	18.9	8.17	0.086
Friends, family or colleagues	15.0	16.5	18.3	10.3	11.48	0.009	15.8	15.2	0.11	0.737	15.6	13.7	0.18	0.669	20.5	15.4	11.6	13.8	15.8	11.68	0.020
**services/systems:**																					
Health services	14.3	10.4	16.9	9.2	17.34	0.001	12.6	12.6	0.00	0.998	12.8	9.0	0.98	0.322	10.5	11.4	12.7	14.2	14.6	4.196	0.38
Social services	27.1	23.9	30.0	16.8	12.60	0.006	27.4	23.0	2.87	0.090	25.0	25.6	0.10	0.926	23.9	25.8	20.6	27.4	27.0	3.59	0.464
Police	24.9	15.1	23.9	13.5	26.79	<0.001	20.9	17.1	3.99	0.046	18.6	24.2	1.32	0.251	14.5	18.5	15.4	20.6	25.3	16.70	0.002
Charities	9.3	7.5	11.8	9.2	5.43	0.143	10.8	8.3	2.72	0.099	9.5	7.6	0.28	0.598	7.2	8.3	8.2	9.3	14.3	11.45	0.022
Government	62.2	62.8	69.7	51.4	34.20	<0.001	65.1	59.7	5.81	0.016	62.7	48.7	6.250	0.012	53.7	62.7	61.1	67.6	66.2	18.51	<0.001
**general advice/info from:**																					
NHS websites	6.7	5.8	10.9	8.3	9.31	0.025	7.1	8.5	0.92	0.339	8.0	6.6	0.16	0.689	4.8	12.0	6.5	5.6	11.1	17.06	0.002
Health apps	27.1	30.6	42.9	37.0	15.78	0.001	35.3	33.8	0.21	0.648	34.4	35.6	0.03	0.873	34.5	39.1	34.9	29.3	33.5	3.89	0.421
General internet sites	28.8	31.2	39.7	38.5	13.68	0.003	35.8	33.3	1.06	0.303	34.6	29.6	0.77	0.380	37.7	34.6	33.0	32.5	33.9	2.30	0.681
Social media	61.5	71.2	77.4	73.2	20.18	<0.001	71.9	71.1	0.11	0.736	72.3	56.9	7.89	0.005	78.4	71.9	68.0	72.3	66.5	11.81	0.019
TV/radio programmes	21.7	25.0	29.0	19.9	11.11	0.011	25.3	24.1	0.34	0.561	24.9	19.4	1.09	0.297	18.5	26.0	28.7	23.6	26.5	11.53	0.021

GPs = General Practitioners. NHS = National Health Service. Apps = Applications. TV = Television.
